# MultiBLUP: improved SNP-based prediction for complex traits

**DOI:** 10.1101/gr.169375.113

**Published:** 2014-09

**Authors:** Doug Speed, David J. Balding

**Affiliations:** UCL Genetics Institute, University College London, London WC1E 6BT, United Kingdom

## Abstract

BLUP (best linear unbiased prediction) is widely used to predict complex traits in plant and animal breeding, and increasingly in human genetics. The BLUP mathematical model, which consists of a single random effect term, was adequate when kinships were measured from pedigrees. However, when genome-wide SNPs are used to measure kinships, the BLUP model implicitly assumes that all SNPs have the same effect-size distribution, which is a severe and unnecessary limitation. We propose MultiBLUP, which extends the BLUP model to include multiple random effects, allowing greatly improved prediction when the random effects correspond to classes of SNPs with distinct effect-size variances. The SNP classes can be specified in advance, for example, based on SNP functional annotations, and we also provide an adaptive procedure for determining a suitable partition of SNPs. We apply MultiBLUP to genome-wide association data from the Wellcome Trust Case Control Consortium (seven diseases), and from much larger studies of celiac disease and inflammatory bowel disease, finding that it consistently provides better prediction than alternative methods. Moreover, MultiBLUP is computationally very efficient; for the largest data set, which includes 12,678 individuals and 1.5 M SNPs, the total analysis can be run on a single desktop PC in less than a day and can be parallelized to run even faster. Tools to perform MultiBLUP are freely available in our software LDAK.

BLUP (best linear unbiased prediction) is perhaps the most widely used tool for prediction of complex traits. Developed in the 1950s as a way to predict random effects in a mixed model (Henderson 1950; Henderson et al. 1959), the advent of genomic selection has further increased its role in animal and plant breeding (Meuwissen et al. 2001; Goddard and Hayes 2007; Habier et al. 2011; Scutari et al. 2013). Recently, as SNP-based heritability analyses have demonstrated the polygenic nature of many human traits (The International Schizophrenia Consortium 2009; Yang et al. 2010), BLUP has also gained popularity among human geneticists, where it is beginning to replace a previous emphasis on sparsity in genome-wide analyses (Makowsky et al. 2011; Yang et al. 2011a; de los Campos et al. 2013).

Central to genetic applications of BLUP is a matrix that encodes genetic similarities between pairs of individuals. It is sometimes called a genomic relatedness matrix, although we consider genomic similarity matrix (GSM) to be more appropriate. The GSM is used to specify the correlation structure of a random effect term in a mixed regression model (“mixed” means that the model includes both fixed and random effects). In the past, the only available measure of genetic similarity was a kinship coefficient computed as a probability of identity by descent in a pedigree, and so a single random effect term sufficed to model genome-wide additive effects. Nowadays genetic similarity can be measured directly, and in many different ways, from genome-wide SNP data. Yet most SNP-based applications of BLUP, referred to as Genomic BLUP or GBLUP, continue to use a single random effect, which corresponds to the unrealistic assumption that all SNP effect sizes are drawn from a common Gaussian distribution. Other authors have attempted to relax the Gaussian assumption, discussed further below, but we believe that the assumption of a common prior distribution is the more important limitation, and so we focus on relaxing that.

We propose MultiBLUP, which generalizes the BLUP model to accommodate multiple random effects. Different SNP classes can be allocated separate random effects, which benefits prediction when the effect-size variance differs markedly across the classes. There are many ways to define SNP classes for which different effect-size distributions may be appropriate, for example: coding, intronic, flanking, and intergenic SNPs; MHC and non-MHC SNPs; SNPs categorized according to conservation across species; and sets of eQTL SNPs for different cell types. Alternatively, we provide Adaptive MultiBLUP which automatically identifies SNP classes with different effect sizes. This adaptive approach begins by dividing the genome into many small regions, which are then merged according to rules intended to identify a small number of genomically contiguous regions, each with effect-size variance distinct from a baseline region comprising the rest of the genome. To ease terminology, we will refer to SNP classes as if defined by genomic regions, although there is no need for the SNPs in a class to be contiguous and classes can overlap. MultiBLUP assigns a random effect to each region, whose correlation structure is determined by a GSM calculated from the SNPs in the region. Here, we focus on GSMs encoding additive genetic effects, but it is possible to include further random effect terms corresponding to dominance or forms of epistasis.

We first apply MultiBLUP to the seven human diseases studied by The Wellcome Trust Case Control Consortium (2007) (WTCCC1). Although relatively small data sets (each comprising ∼5000 individuals recorded for 280,000 SNPs), these allow us to demonstrate the advantages of MultiBLUP over a range of diseases with different genetic architectures. For rheumatoid arthritis and Type 1 diabetes, we show improvements in predictive accuracy from assigning distinct random effects to SNPs within and outside the major histocompatibility complex (MHC), because for these two traits, MHC SNPs tend to have larger effects. Compared to BLUP, genetic risk scores (Wray et al. 2007), stepwise regression (Purcell et al. 2007), and Bayesian sparse linear mixed models (BSLMM) (Zhou et al. 2013), we find Adaptive MultiBLUP to be the overall top performing method, regardless of whether we measure the accuracy of predicted phenotypic values by correlation, mean squared error, median absolute error, or area under curve (AUC). Moreover, Adaptive MultiBLUP requires only a fraction of the computational time and memory resources of BSLMM, the second best performing method.

We next tackle larger data sets—for celiac disease (∼15,000 individuals, 200,000 genotyped SNPs) and inflammatory bowel disease (13,000 individuals, 1.5 M imputed SNPs). Genetic screening of patients is routinely used to guide diagnosis and hence treatment for celiac disease (Husby et al. 2012; Abraham et al. 2014); and so for this trait, improved prediction can have immediate impact. Due to the size of these data sets, it is not feasible to run stepwise regression or BSLMM; in contrast, Adaptive MultiBLUP requires <4 GB of memory, and completes in ∼6 h for celiac disease and 24 h for inflammatory bowel disease. Again, we find Adaptive MultiBLUP to be greatly superior to both standard BLUP and genetic risk scores.

Lastly, we consider 139 phenotypes from the Wellcome Trust Heterogeneous Stock mouse collection, where individuals are highly related as is typical in plant and animal breeding. Although there is little difference between the performance of BLUP, BSLMM, and Adaptive MultiBLUP, our results demonstrate that MultiBLUP can also be used when the data set contains high levels of structure.

The tools required to perform MultiBLUP prediction are freely available in our software LDAK.

## Results

The WTCCC1 data consist of two control and seven case data sets for bipolar disorder (BD), coronary artery disease (CAD), Crohn’s disease (CD), hypertension (HT), rheumatoid arthritis (RA), Type 1 diabetes (T1D), and Type 2 diabetes (T2D). Our quality control (see Methods; Supplemental Fig. 1) removed individuals inferred to be of non-Caucasian ancestry and reduced the number of genotypes to ∼280,000. For our simulation study, we use the 2959 control individuals and the 47,546 SNPs from chromosomes 1 and 2. For the analysis of observed phenotypes, we combined in turn the 2959 controls with the ∼2000 case individuals for each of the seven traits and used all SNPs.

For celiac disease, we use the data of Dubois et al. (2010). Individuals were sourced from five cohorts, labeled according to country of origin (UK1, UK2, Finland, Netherlands, and Italy). After quality control, 15,283 individuals and 190,948 SNPs remained (see Methods; Supplemental Fig. 2). For inflammatory bowel disease, we combine data from WTCCC and the National Institute of Diabetes and Digestive and Kidney Disease (NIDDK): Starting with the 1916 Crohn’s disease cases from WTCCC1, we add 8033 individuals from WTCCC2 (5200 population controls and 2833 ulcerative colitis cases) and 2788 individuals from the NIDDK (813 cases for Crohn’s disease and 947 matched controls, and 1028 ulcerative colitis cases). As genotyping was performed using multiple SNP arrays, we first imputed using IMPUTE2 against the 1000 Genome reference panel (The 1000 Genomes Project Consortium 2010; Howie et al. 2011). After quality control, 12,678 individuals and 1,487,824 SNPs remained (see Methods; Supplemental Fig. 3).

The mouse data set consists of 1940 heterogeneous stock mice descended from eight founder lines (Valdar et al. 2006). After quality control, there were 8516 SNPs across 19 autosomes. The equal-tailed 95% interval for the kinship coefficients is [−0.11, 0.24], indicating high levels of relatedness (the corresponding interval for the WTCCC1 data is [−0.01, 0.01]). For our simulation study, we use all individuals and SNPs. For the real data analysis, of the >150 traits available, we focus on the 139 quantitative traits that have measurements available for at least 1300 mice and a coefficient of kurtosis less than six (the kurtosis of the Gaussian distribution is three). The chosen traits, which all had a coefficient of skewness <1.5 (the Gaussian distribution has skewness zero), span behavioral, hematological, biochemical, and disease-related phenotypes. For many of the traits, the phenotypic values are strongly correlated with cage ID. Therefore, when performing cross-validation, we ensure that individuals in the same cage remain in the same fold.

### Simulation study

First, we demonstrate the potential of MultiBLUP in a simple, albeit unrealistic, setting in each of two data sets (WTCCC1 and mouse). We divide the SNPs into five distinct regions and simulate quantitative phenotypes in which each region contributes a specified heritability. We consider three scenarios: (1) The five regions contribute equally to heritability; (2) regions contribute to heritability in the ratios 1:2:3:4:5; and (3) only Region 5 contributes to heritability. In each region that contributes to heritability, we assign additive genetic effects to 20 random SNPs, with effect sizes drawn from a Gaussian distribution with mean zero and variance chosen to achieve the required heritability. When applying BLUP, we used a single GSM computed as average allelic correlations across all five regions. With MultiBLUP, we used five GSMs, each calculated in the same way but using SNPs from only one of the regions. For both BLUP and MultiBLUP, we divided individuals between training and test sets in the ratio 5:1, then measured prediction performance of models fitted on the training set by the correlation between simulated and predicted phenotypes in the test set.

**Figure 1.**
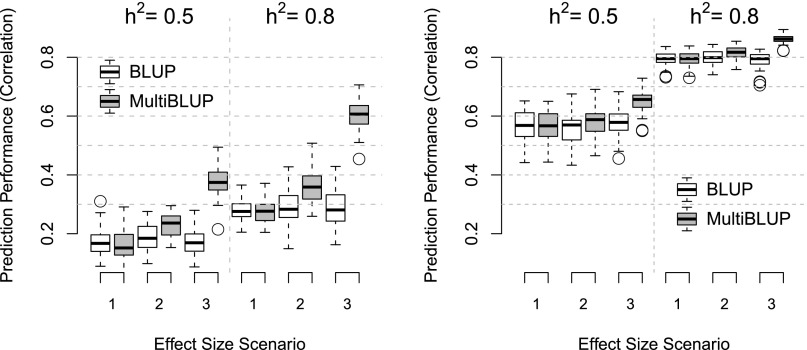
Prediction performance of BLUP and MultiBLUP on simulated quantitative traits. The two plots correspond to unrelated humans (*left*) and related mice (*right*). They show across 50 repetitions the correlation between predicted and observed phenotypes in the test set for BLUP (white boxes) and MultiBLUP (shaded boxes). The *x*-axis indexes the simulation scenarios, with increasing heterogeneity of effect sizes across the five regions. Here, MultiBLUP uses five GSMs, one for each region. Within each plot, the true (simulated) heritability is 0.5 (*left* half) or 0.8 (*right* half).

Figure 1 shows that for both data sets, the two methods perform similarly for Scenario 1, indicating little disadvantage to assuming the more general MultiBLUP model when it is not needed. The performance of BLUP does not improve with increasing concentration of causal variation in Scenarios 2 and 3, whereas MultiBLUP does exploit the heterogeneity of effect sizes to improve prediction, dramatically so for the WTCCC1 data. Prediction performance is good in all scenarios for the mice, because close relatedness implies that almost all causal variants are tagged, but even here the improvement of MultiBLUP is noticeable. We repeated the analysis when heritability was distributed across all SNPs, rather than only a selected 20, and observed similar results (Supplemental Fig. 4). As well as prediction performance, we also measure genomic selection performance, which is the accuracy of estimation of the sum of the random effects. This is known in animal and plant genetics as the “breeding value,” and represents the phenotypic value after discounting environmental noise. MultiBLUP also provides better genomic selection performance than BLUP (Supplemental Fig. 5).

### WTCCC1 data

For the real phenotypes, we evaluate prediction methods using 10-fold cross-validation. When comparing methods applied to binary outcomes, it suffices to treat case/control status as a continuous variable (cases 1, controls 0), because there exists a linear relationship between prediction performance on the observed and underlying liability scales (Dempster and Lerner 1950; Yang et al. 2011a; Zhou et al. 2013). Moreover, this permits us to minimize any effects of confounding by first regressing case/control status on sex and the first 20 principal component axes.

Columns 1–4 of Table 1 report the performance of BLUP, genetic risk scores, stepwise regression, and BSLMM (see Methods for details of parameter choices). Of the first three methods, there is no clear winner: Stepwise regression performs best for RA and T1D, the two traits with strongest marginal associations, while BLUP and genetic risk scores fare better for the more polygenic traits BD and HT. In contrast, BSLMM, whose model allows for both sparsity and shrinkage, performs well regardless of the genetic architecture of the trait and overall is the best of the current methods.

**Table 1.**
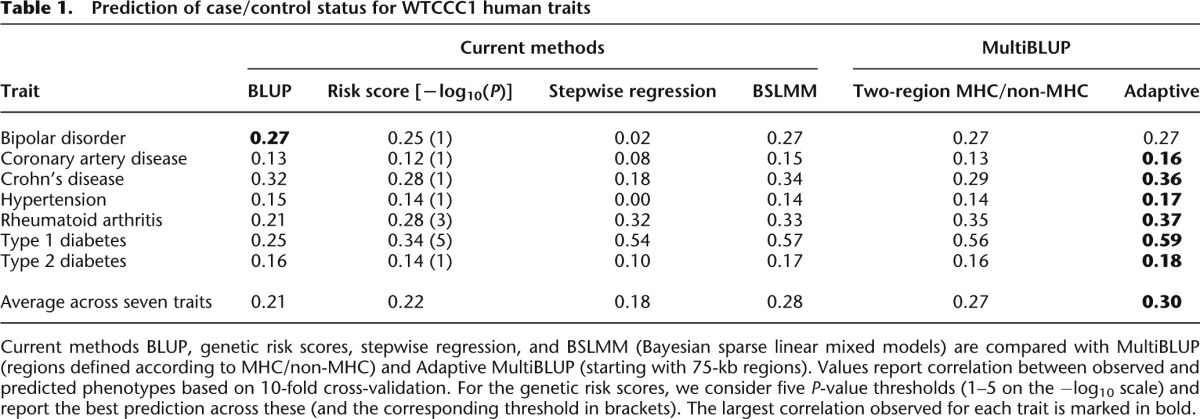
Prediction of case/control status for WTCCC1 human traits

As a first demonstration of MultiBLUP, we consider two regions: one corresponding to the extended MHC (chr. 6: 25–34 Mb) and one to all other SNPs (Table 1, Column 5). This relatively simple change to the BLUP model leads to greatly improved prediction for the autoimmune traits RA and T1D (correlation 0.35 and 0.56, respectively, compared to 0.21 and 0.25 for BLUP). Supplemental Figure 6 shows Manhattan plots for each trait, from which the enhanced role of the MHC for RA and T1D can be seen. Supplemental Table 1 shows heritability estimates for the MHC and non-MHC regions and how much each contributes to prediction. Separating SNPs according to MHC improves prediction when the MHC harbors substantial heritability; conversely, high heritability can be attributed to non-MHC SNPs without this contributing much to prediction, because a single SNP effect-size variance is inadequate for a large, heterogeneous region.

The SNPs in each region need not be contiguous. To illustrate this, we partition the genome into two regions according to eQTL status. For this application, we classify “eQTL SNPs” as those associated (*P* < 10^−10^) with changes in expression levels for at least one gene, according to Curtis et al. (2012). Using this threshold, ∼5% of SNPs are classified as eQTLs. Compared to BLUP, we achieve improved prediction for RA and T1D (see Supplemental Table 2), indicating that for these traits the eQTL SNPs tend to have a larger influence than the non-eQTL ones. Similarly, MultiBLUP regions can overlap, which we illustrate for CD by constructing regions based on two pathways (IL-2 receptor beta chain in T cell activation and IL12 pathway) and two genes (*NOD2* and *IL23R*), all of which have shown association with the trait in at least two data sets other than the WTCCC1 (Agura et al. 2001; Hugot et al. 2001; Duerr et al. 2006; Wang et al. 2009; Ballard et al. 2010). A fifth region contains all other SNPs. Prediction is slightly improved compared to BLUP (correlation 0.319 versus 0.316) (see Supplemental Table 3)

Rather than rely on prior information to define SNP regions, MultiBLUP can be run adaptively, starting with many small genomic regions which are then merged as described below and in Methods. For each of the WTCCC1 traits, we begin by dividing the genome into ∼68,000 regions of size 75 kb (on average, eight SNPs), with a 37.5-kb overlap between neighboring regions. Although our aim is to identify regions with above-average effect-size variance, because the individuals are predominantly unrelated, most effect-size variances will be very small; therefore, it suffices (and is much faster) to test instead whether each effect-size variance is nonzero. Each region with *P* < 10^−6^ is merged with any neighboring region with *P* < 10^−2^. At the end of this process, all remaining regions are merged into a background region. For the highly polygenic traits BD, CAD, HT, and T2D, this process generates one to two regions (including the background region); for CD, RA, and T1D, we find on average seven, five, and eight regions, respectively (see Supplemental Fig. 7). Overall, we find Adaptive MultiBLUP (Table 1, Column 6) to be the best-performing method; it ranks first for six of the seven traits and is only narrowly beaten by BLUP for BD. Adaptive MultiBLUP remains top if instead we measure prediction accuracy according to mean squared error, median absolute error, or AUC (Supplemental Table 4).

### Celiac disease and inflammatory bowel disease

For these two traits, the sizes of the data sets make it infeasible to run stepwise regression or BSLMM, so we restrict comparison to BLUP, genetic risk scores, and Adaptive MultiBLUP (starting as before with overlapping 75-kb regions). Increasing the sample size improves the resolution of Adaptive MultiBLUP; for example, for inflammatory bowel disease, the method identifies on average 27 distinct local regions (Supplemental Fig. 8). We again find Adaptive MultiBLUP to be the best performing method (Table 2; Supplemental Table 5). For celiac disease, we additionally consider a linear prediction model constructed from 77 susceptibility SNPs: 6 SNPs tagging four human leukocyte antigen (HLA) haplotypes (Monsuur et al. 2008) and 71 SNPs based on Romanos et al. (2014). For this model, the average correlation is 0.40 and the average AUC is 0.78 (see Supplemental Table 6), demonstrating here that it is better to incorporate genome-wide SNP data than use only top associated SNPs. Unlike celiac disease, genetic testing is not yet routinely used for inflammatory bowel disease, but its potential for distinguishing subtypes has been discussed. For example, although the low prevalence of Crohn’s disease makes prediction at the population level difficult, genetic data could aid in the diagnosis of patients presenting with abdominal pain, diarrhea, and weight loss (Jostins and Barrett 2011), and the case for this is strengthened by the improved predictive accuracy of MultiBLUP.

**Table 2.**
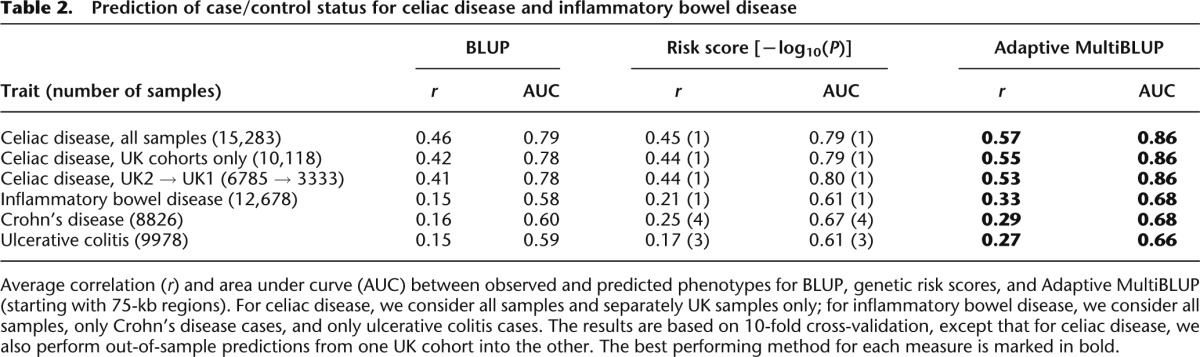
Prediction of case/control status for celiac disease and inflammatory bowel disease

### Mouse data

Supplemental Figure 9 shows the performance of BLUP, genetic risk scores, BSLMM, and Adaptive MultiBLUP across the 139 mouse phenotypes. Again, we start Adaptive MultiBLUP with overlapping 75-kb regions, but owing to the smaller size of the mouse genome, relax the initial significance threshold to *P* < 5 × 10^−6^. We find that genetic risk scores is by far the worst performing method (average correlation 0.27), because the basic single-SNP association test it uses copes poorly with the structure present in the data set. Overall, the performances of BLUP, BSLMM, and Adaptive MultiBLUP are very similar (average correlations 0.335, 0.336, and 0.336, respectively), with different methods performing best for different phenotypes. As explained below, the advantage of BSLMM and Adaptive MultiBLUP relative to BLUP comes from being able to identify individual causal loci with relatively strong influence on the phenotype; however, the high levels of relatedness and low SNP density present in the mouse data will generally make this difficult.

In this application, Adaptive MultiBLUP is slowed down due to the high levels of relatedness; despite there being fewer than 2000 individuals, it now takes ∼3 h to analyze each phenotype, approximately as long as BSLMM. This is because when deciding how to divide SNPs into regions, the shortcut used for the human data (testing whether each initial region has effect-size variance greater than zero) is no longer valid. However, this step is parallelizable, and we anticipate that it can be made orders of magnitude faster by implementing algorithmic speed-ups similar to those proposed by Listgarten et al. (2012).

### Comparison between Adaptive MultiBLUP and BSLMM

The prediction models used by Adaptive MultiBLUP and BSLMM have much in common. For both methods, a relatively small number of SNPs are used to capture the contributions of distinct causal loci, whereas the majority of SNPs influence the prediction model only through a polygenic term (in Adaptive MultiBLUP, this corresponds to the background region). The major difference is that in BSLMM, each causal locus is typically represented by only one or two SNPs, whereas a local region in Adaptive MultiBLUP will generally include multiple SNPs. The former approach might be expected to perform better when a reasonably strong causal variant is well tagged by a single SNP, but even then, prediction is unlikely to suffer much by including some extra SNPs. In contrast, when the causal variant is difficult to detect through single-SNP analysis, either because it is not well tagged or has effect size too weak, or when a local region contains two or more causal variants, using multiple SNPs can provide improved prediction.

This would suggest that the accuracy of Adaptive MultiBLUP, and the potential to outperform BSLMM, will tend to increase with SNP density. Adaptive MultiBLUP was noticeably better than BSLMM for the WTCCC1 data sets, which have on average eight SNPs per 75 kb, and we predict that had it been feasible to apply BSLMM to the inflammatory bowel disease data set (44 SNPs per 75 kb), the gap between the two methods would have been even larger. Conversely, for the mouse data the genotyping is much more sparse (most 75-kb regions contain only a single SNP), and Adaptive MultiBLUP no longer has an advantage. The difference between prediction models also explains the disparity in computational demands. The BSLMM model has one effect size for each SNP, plus additional parameters, whose values are estimated using Markov Chain Monte Carlo (MCMC). In contrast, Adaptive MultiBLUP has many fewer parameters (one variance component for each region, plus one for the environmental noise term); this allows the prediction model to be fitted deterministically, which is much faster than using MCMC and avoids issues of parameter convergence. Similarly, the memory demands of Adaptive MultiBLUP are much lower. Both methods must store a (genome-wide) GSM, but whereas BSLMM must also read in the entire data set, Adaptive MultiBLUP requires only those SNPs included in local regions (which is typically a small fraction of the total number of SNPs). For this reason, although it was not possible to apply BSLMM to the celiac or inflammatory bowel disease data sets, Adaptive MultiBLUP could realistically be run on even larger data sets: up to around 50,000 individuals with full-genome sequence data.

## Discussion

We have presented MultiBLUP, a powerful and efficient method for prediction of complex traits from genome-wide SNP data. The statistical model underlying BLUP was developed for use with kinship coefficients derived from pedigrees, but SNP data allows additional flexibility that has not previously been exploited. Specifically, the BLUP model assumes that SNP effect sizes have the same distribution for all SNPs. MultiBLUP generalizes this model by introducing multiple random effects, allowing different effect-size variances for different classes of SNPs. The SNP classes used in MultiBLUP can be identified using prior information, for example, about genes and pathways relevant to the trait or other functional annotation of SNPs. Alternatively, Adaptive MultiBLUP can automatically identify SNP regions with different effect-size variances. In fact, there is no need for the correlation structure of MultiBLUP’s random effects to be defined by SNPs; its prediction model can integrate multiple sources of data including copy number variants, measures of gene expression or methylation, and pedigree information.

Previous attempts to generalize the BLUP model have mainly focused on weakening the Gaussian assumption for SNP effect sizes, which has been rightly criticized because of the “thin tails” property of the Gaussian distribution. The *t*, double-exponential and normal-exponential-gamma distributions have been suggested as alternatives, as well as mixture distributions that allow many SNPs to have zero or negligible effect (for review, see Zhou et al. 2013). It is not practical to compare MultiBLUP with all rival methods; so in addition to BLUP, genetic risk scores and stepwise regression, we chose BSLMM, an approach that seeks to incorporate ideas from many of the BLUP generalizations and that has been shown to outperform a number of alternative methods (Zhou et al. 2013). The advantage of BSLMM over BLUP, genetic risk scores, and stepwise regression was apparent in our analyses of the WTCCC1 data, but we found it to be inferior to Adaptive MultiBLUP for all traits, with Adaptive MultiBLUP requiring only 10% of the computation time and 5% as much memory as BSLMM. For the much larger celiac disease and inflammatory bowel disease data sets, we showed that MultiBLUP continued to outperform the computationally feasible alternatives, whereas the mouse data demonstrated that MultiBLUP can also perform well for structured data sets.

Consortia now exist for a wide variety of traits, combining data across tens of thousands of patients, while initiatives such as the 100,000 Genomes Project (http://www.genomicsengland.co.uk) are set to recruit individuals in even larger numbers. At the same time, with next generation sequencing becoming more widely available and with our ability to interrogate other sources of information (for example, transcriptomic and epigenomic) constantly improving, the number of predictors available will continue to increase. With much of the algorithm parallelizable, there is essentially no limit to the number of predictors that MultiBLUP can analyze. Instead, the runtime of MultiBLUP depends primarily on the number of individuals because this affects how long it takes to estimate the variance components. The current implementation of Adaptive MultiBLUP can analyze 50,000 individuals, and we expect algorithmic advances to lead to increases in this number. We also envisage a meta-analysis version of MultiBLUP, in which prediction models are constructed locally and then combined, allowing MultiBLUP to be used by meta-analysis consortia, where data cannot be shared centrally.

SNP-based prediction of phenotype is central to genomic selection, which is revolutionizing animal and plant breeding. For humans, prediction is more challenging because we are largely outbreeding, which leads to low levels of relatedness in most populations. Moreover, the binary nature and low prevalence of many disease phenotypes imply that prediction of disease onset is typically not useful in a general population. However, prediction of disease state from genotype already has clinical utility in individuals selected to be of high risk on the basis of nongenetic risk factors. Moreover, where decisions about treatment options are already based on risk factor scores, genomic information can contribute substantially to improved decision making at the population level, even when individual predictions are imprecise. As the costs of genome-wide genotyping continue to fall, additional clinical uses of genomic information for prediction of traits in humans will be found, for example, to generate more realistic, individual-specific baselines from which to assess environmental impacts in population health studies. These will allow additional benefits to be obtained from the superior predictions of MultiBLUP.

## Methods

The usual BLUP model assumes that ***Y***, the vector of phenotypic values for *n* individuals, is influenced by random effects ***g*** (genetic) and ***e*** (environmental) via
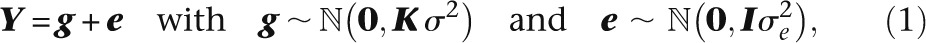
where ***K*** is a GSM specifying the correlation structure of ***g***, ***I*** is an *n* × *n* identity matrix, and *σ*^2^ and 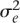 are variances (for simplicity, fixed effects have been ignored). A common SNP-based GSM is allelic correlations averaged over SNPs (Astle and Balding 2009):
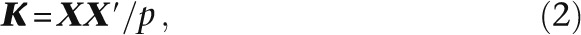
where ***X*** is a matrix of (normalized) SNP genotypes, ***X***′ is its transpose, and *p* is the number of SNPs. If Equation 2 holds, then Equation 1 can be expressed as a linear regression with random coefficients (Hayes et al. 2009):
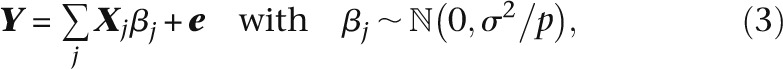
where ***X***_*j*_ denotes the *j*th column of ***X***, and *β*_*j*_ is a measure of effect size for the *j*th SNP.

MultiBLUP extends Equation 1 to include random effects ***g***^1^, …, ***g***^*M*^, with correlation structures specified by ***K***^1^, …, ***K***^*M*^, and the corresponding variances 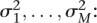:
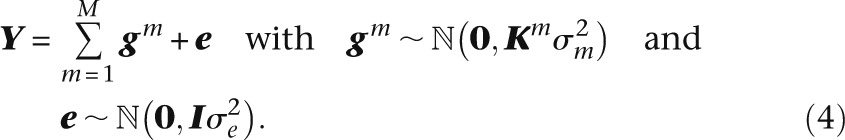
When each ***K***^*m*^ is of the form of Equation 2 for a matrix ***X***^*m*^ with columns corresponding to a set of SNPs *R*_*m*_ of size *p*_*m*_, the corresponding random regression model is
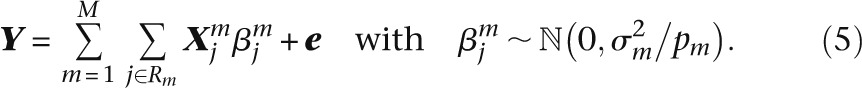
As with BLUP, the key computational step of MultiBLUP is the estimation of the variance parameters 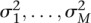 and 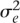. This can be achieved using (a generalized version of) REML (Corbeil and Searle 1976), which maximizes the log likelihood:
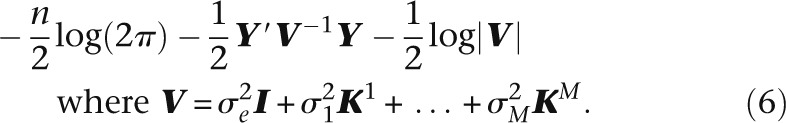
MultiBLUP computes 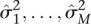 and 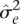, estimates of the variance components, using average information REML (Gilmour et al. 1995; Lee and van der Werf 2006). If the total proportion of variance explained by a kinship matrix is below 0.01% for two consecutive iterations, its contribution is set to zero; there is no limit on how many variance terms can be set to zero, allowing MultiBLUP to be run with very many regions.

MultiBLUP includes two computational optimizations to reduce memory usage and time requirements. First, when a region contains fewer SNPs than the number of individuals, the corresponding GSM, ***K***^*m*^ = ***X***^*m*^(***X***^*m*^)′/*p*_*m*_, is computed on-the-fly, meaning that only ***X***^*m*^, rather than ***K***^*m*^, need be stored. Second, in each iteration, the most time-consuming step is inverting ***V***; however, this process can be sped up whenever at most one GSM has full rank, and the total number of SNPs contributing to the remaining GSMs is less than the number of individuals (which is generally the case for Adaptive MultiBLUP). Suppose that ***K***^1^ is the GSM with full rank, then it can be decomposed as ***K***^1^ = ***UEU***′, where ***U*** is orthogonal and ***E*** diagonal, and therefore 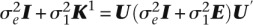. The Woodbury Matrix Identity states that
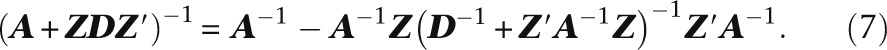


Let 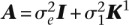, concatenate the remaining regional SNP matrices into ***Z*** = [***X***^2^ ***X***^3^ … ***X***^*M*^], and construct the diagonal matrix ***D*** with diagonal elements consisting of 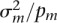 repeated *p*_*m*_ times, for *m* = 1, …, *M*. Then ***V*** is in the form required to apply Equation 7. Because 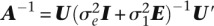 and ***D*** is diagonal, the only inversion required is of the lower-dimensioned matrix (***D***^−1^ + ***Z***′***A***^−1^***Z***)^−1^. Moreover, by keeping ***V***^−1^ in the form ***UWU***′, it is possible to carry out the REML iterations without computing ***V***^−1^ explicitly, avoiding the need to multiply matrices of size *n* × *n*. Additionally, this implementation avoids problems caused by local region kinship matrices being low-rank and therefore not invertible.

### Predicting phenotypes

Suppose that phenotypes are recorded for individuals indexed by the set *S*, and we wish to predict those for individuals in the set *T*. In addition to estimating the variance parameters, REML also obtains 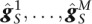, estimates of the genetic random effects for individuals in *S*. To predict phenotypes for individuals in *T*, we estimate 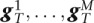 by their expected values given 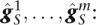:
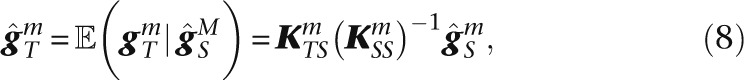
where 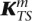 and 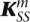 are submatrices of ***K***^*m*^ defined by the subscripts. We can then predict phenotypes for individuals in *T* via 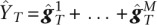. When the GSM takes the form of Equation 2, we have
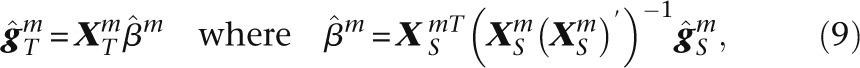
so that 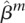 is the vector of effect sizes for SNPs in ***X***^*m*^. When ***K***^*m*^ corresponds to a local region, it is typically not invertible, so instead we use
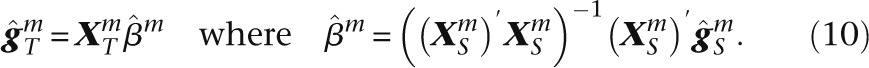
Using Equations 9 and 10 is often more convenient than using Equation 8 because then phenotypes for test individuals can be predicted without needing to refer to data for training individuals.

### Adaptive MultiBLUP

If regions *m* and *m*′, of sizes *p*_*m*_ and *p*_*m*′_, have equal effect-size variances (i.e., 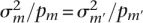), then Equation 5 is unaffected by merging the two regions or, equivalently, replacing ***g***^*m*^ and ***g***^*m*′^ in Equation 4 with a single random effect with correlation structure (*p****K***_*m*_ + *p*′***K***_*m*′_)/(*p* + *p*′). Therefore, our adaptive strategy starts by dividing the genome into genomically local SNP regions, then testing for each region whether its effect-size variance is significantly greater than that for all other regions combined. The formal test for Region *m* is performed by calculating *l*_0_, the maximum value of Equation 6 using a single GSM
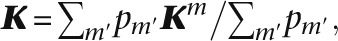
and *l*_1_, the maximum value using two GSMs: ***K***^*m*^ and its complement
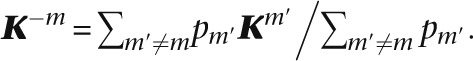
A *P*-value is obtained by comparing the test statistic 2(*l*_1_ − *l*_0_) to a *χ*^2^(1) distribution. When levels of relatedness are low, it suffices to instead test whether the contribution to heritability from each region is significantly different from zero, using highly efficient computations similar to those outlined by Listgarten et al. (2013). The starting region size (we chose 75 kb for the human data) is intended to be small enough to separate distinct causal loci, but in case a causal locus spans multiple 75-kb regions, we then merge adjacent significant regions as described above. To test sensitivity for the WTCCC1 data, we additionally ran Adaptive MultiBLUP starting with 37.5 and 150-kb regions, or using significance thresholds *P* < 10^−5^ and *P* < 10^−7^ (instead of the Bonferroni-derived *P* < 10^−6^); in all cases we observed little difference in prediction performance, and Adaptive MultiBLUP remained the best performing method (see Supplemental Table 7).

Equation 4 is the same as that used by genome partitioning, a method for estimating the variance explained by subsets of SNPs (Yang et al. 2011b). The advantage of MultiBLUP over BLUP arises when relatively large fractions of phenotypic variance can be assigned to relatively small SNP classes. However, it is important to bear in mind that the focus of MultiBLUP is to obtain the prediction model 
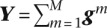
 (or equivalently 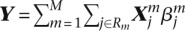); the estimates 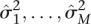 will only accurately reflect variance explained when individuals are distantly related and when the decision of how to construct each ***K***^*m*^ was made a priori, rather than adaptively, based on the data.

### Data quality control

All analyses used only autosomal SNPs. For the WTCCC1 data (http://www.wtccc.org.uk), we filtered to remove population outliers identified through principal component analysis (Supplemental Fig. 1), after which 2959 controls remained and each of the case/control studies were left with between 4859 and 4928 individuals. Then we removed SNPs with either minor allele fraction (MAF) < 0.01 or call-rate (CR) < 0.995, or *P* < 0.05 from either a test for Hardy-Weinberg equilibrium (HWE) or differential missingness between cases and controls, after which studies contained between 270,319 and 284,913 SNPs. Even subtle differences in population between cases and controls can lead to artificial gains in prediction. To guard against this, we first regressed disease status on sex plus the top 20 principal component axes, then used the (continuous-valued) residuals for subsequent analyses. A potential drawback of this approach is that any true causal signal contained within the top axes is discarded and so is unable to contribute toward prediction; however, as population stratification is likely to benefit most methods whose prediction models contain very many SNPs, we thought it better to err on the conservative side. By way of comparison, we instead regressed disease status on sex and two ancestry axes derived from the HapMap reference panel (The International HapMap Consortium 2003) observing slightly higher prediction performance for the WTCCC1 traits (see Supplemental Table 7), suggesting that the true prediction potential lies somewhere in between these two sets of values.

For the celiac disease data, the initial quality control steps are described in Dubois et al. (2010). Principal component analysis indicated that the data set was sufficiently homogeneous (Supplemental Fig. 2), so we retained all 15,283 individuals, but removed SNPs with MAF < 0.01, CR < 0.995, or HWE *P* < 0.05, after which 190,948 remained. For inflammatory bowel disease, the data came from five cohorts: 1916 Crohn’s disease cases from WTCCC1; 5200 controls and 2833 ulcerative colitis cases from WTCCC2; 813 Crohn’s disease cases and 947 matched controls; and 1028 ulcerative colitis cases from NIDDK (http://www.niddk.nih.gov). Separately for each cohort, we first removed outlying samples based on principal component analysis (Supplemental Fig. 3), and SNPs with MAF < 0.01, CR < 0.95, or HWE *P* < 10^−6^, then imputed against the 1000 Genome reference panel using IMPUTE2 (The 1000 Genomes Project Consortium 2010; Howie et al. 2011). Then we combined samples, filtering out SNPs with (expected) MAF < 0.01, (expected) CR < 0.995, or IMPUTE2 Info Score <0.98, and finally excluding 213 individuals who appeared to be duplicates (estimated kinship >0.7 with another individual in the data set): 12,678 individuals and 1,487,824 SNPs remained.

For the mouse data (downloaded from http://mus.well.ox.ac.uk/mouse), no individuals were excluded, but SNPs were removed if they had MAF < 0.01, HWE *P* < 10^−4^, or call-rate <0.99. Each of the supplied phenotypes had been preadjusted for marginally significant covariates, such as age, sex, and body weight, and when performing 10-fold cross-validation, we ensured that mice in the same cage were kept in the same fold.

### Genetic risk scores

We constructed a linear predictor 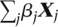 using all SNPs achieving *P*-values from marginal association analysis below a specified threshold, with effect sizes estimated from the same analysis. We considered five threshold values (1–5 on the −log_10_ scale), expecting higher *P*-value thresholds to provide better prediction for more highly polygenic traits and vice versa. Prediction from genetic risk scores can be impaired due to high levels of linkage disequilibrium; so for the human data we repeated the analysis having first pruned to obtain a subset of SNPs in approximate linkage equilibrium. Results were noticeably different only for the inflammatory bowel disease data set, which uses imputed genotypes; so for this trait, we instead report results from the pruned analysis. Because genetic risk scores estimate SNP effect sizes independently, the method is not expected to perform well when judged according to mean squared error or median absolute error, and this proved to be the case for all traits.

### Stepwise regression

We performed multiple runs of single-SNP association analysis, each time conditioning on the SNPs already selected and adding the most strongly associated SNP to the model. We stopped when no SNP was (conditionally) significant at *P* < 10^−6^, then estimated coefficients for the selected SNPs using least squares. For BD, CAD, HT, and T2D, the average model size was between zero and two SNPs, whereas for CD, RA, and T1D, the average model size was seven, six, and 17 SNPs, respectively.

### BSLMM

We ran BSLMM with the parameters at their default values, meaning that the first 100,000 MCMC iterations were discarded, then posterior estimates were obtained from the next 1,000,000. For both the human and mouse data, we used a standardized kinship matrix (option -gk 2), matching the way GSMs were computed for BLUP and MultiBLUP.

### Computing resources

The first step in MultiBLUP is to compute one or more GSMs. When these represent allelic correlations, the time required scales approximately linearly in the total number of SNPs and quadratically in the number of individuals; for example, with optimized code (Gray et al. 2012), this step took ∼15 h for the inflammatory bowel disease data (∼13,000 individuals, 1.5 M SNPs), and is readily parallelized. For Adaptive MultiBLUP, each initial region must be tested, which when individuals are predominantly unrelated is very fast (about 1 h for the inflammatory bowel disease data), but slower when individuals are highly related (about 3 h for the mouse data); however, this step can also be parallelized. The time to estimate the variance terms scales approximately quadratically in the number of individuals, taking ∼5 h for the inflammatory bowel disease data. The memory required by MultiBLUP scales quadratically with the number of individuals and, if each GSM has full rank, linearly with the number of random effects (e.g., with two full-rank GSMs, MultiBLUP requires about twice the memory of BLUP); however, GSMs corresponding to small subsets of SNPs can be computed on-the-fly, meaning that Adaptive MultiBLUP typically requires only slightly more memory than BLUP (∼4 Gb for the inflammatory bowel disease data).

### Software availability

The tools required to apply MultiBLUP are freely available in our software LDAK (http://www.ldak.org).
